# The Seasonal Patterns, Ecological Function and Assembly Processes of Bacterioplankton Communities in the Danjiangkou Reservoir, China

**DOI:** 10.3389/fmicb.2022.884765

**Published:** 2022-06-15

**Authors:** Zhao-Jin Chen, Yong-Qi Liu, Yu-Ying Li, Li-An Lin, Bao-Hai Zheng, Ming-Fei Ji, B. Larry Li, Xue-Mei Han

**Affiliations:** ^1^International Joint Laboratory of Watershed Ecological Security and Collaborative Innovation Center of Water Security for Water Source Region of Middle Route Project of South-North Water Diversion in Henan Province, School of Water Resource and Environmental Engineering, Nanyang Normal University, Nanyang, China; ^2^Ecological Complexity and Modelling Laboratory, Department of Botany and Plant Sciences, University of California, Riverside, Riverside, CA, United States; ^3^Ministry of Education Key Laboratory for Ecology of Tropical Islands, College of Life Sciences, Hainan Normal University, Haikou, China

**Keywords:** Danjiangkou Reservoir, bacterioplankton communities, seasonal variations, ecological network analysis, neutral model

## Abstract

As the water source for the Middle Route Project of the South-to-North Water Diversion Project (MR-SNWD) of China, the Danjiangkou Reservoir (DJR) is in the process of ecosystem reassembly, but the composition, function, and assembly mechanisms of bacterioplankton communities are not yet clear. In this study, the composition, distribution characteristics and influencing factors of bacterioplankton communities were analyzed by high-throughput sequencing (HTS); PICRUSt2 was used to predict community function; a molecular ecological network was used to analyze bacterioplankton interactions; and the assembly process of bacterioplankton communities was estimated with a neutral model. The results indicated that the communities, function and interaction of bacterioplankton in the DJR had significant annual and seasonal variations and that the seasonal differences were greater than that the annual differences. Excessive nitrogen (N) and phosphorus (P) nutrients in the DJR are the most important factors affecting water quality in the reservoir, N and P nutrients are the main factors affecting bacterial communities. Season is the most important factor affecting bacterioplankton N and P cycle functions. Ecological network analysis indicated that the average clustering coefficient and average connectivity of the spring samples were lower than those of the autumn samples, while the number of modules for the spring samples was higher than that for the autumn samples. The neutral model explained 66.3%, 63.0%, 63.0%, and 70.9% of the bacterioplankton community variations in samples in the spring of 2018, the autumn of 2018, the spring of 2019, and the autumn of 2019, respectively. Stochastic processes dominate bacterioplankton community assembly in the DJR. This study revealed the composition, function, interaction, and assembly of bacterioplankton communities in the DJR, providing a reference for the protection of water quality and the ecological functions of DJR bacterioplankton.

## Introduction

The Danjiangkou Reservoir (DJR) is the water source for the MR-SNWD of China (Liu et al., 2018). To meet the demand of MR-SNWD water transfer, the water level in the DJR was raised from 157 m to 170 m by increasing the height of the dam in 2013, and the inundation area was increased by 302.5 km^2^ (Shu et al., 2017). The water flow direction in the DJR changed dramatically, from the original outflow from the Danjiangkou Dam to the diversion of water at the head gate of the Taocha Channel to the north, eventually reaching Beijing and Tianjin. Therefore, the DJR ecosystem is in the process of reconstruction, providing an excellent location for the study of bacterioplankton composition, function, interaction and community assembly in large reservoirs (Pan et al., 2018).

Bacterioplankton, as the main component of the aquatic community, plays an important role in the matter cycling in freshwater bodies and drive chemical element cycles in the entire ecosystem (Ducklow et al., 1986; Grossart, 2010). In addition, bacterioplankton are sensitive to water quality and environment changes: changes in factors such as the content of nutrients in different forms and the physicochemical indicators of the freshwater body will influence bacterioplankton community structure (Morris and Lewis, 1992; Mohapatra et al., 2020). Therefore, analyzing variations in bacterioplankton community structure in a reservoir and the difference in the degree of response to the physical and chemical properties can well reflect the water environment of the reservoir and can be used as an important indicator for analyzing the health of the reservoir ecosystem. Currently, few studies have been carried out on the composition of the bacterioplankton communities in the DJR and the associated influencing factors. In our previous study, HTS was employed for bacterioplankton community composition and influencing factors in the DJR, found that the bacterioplankton community were composed of 27 phyla and 336 genera and that TN, pH, COD, and COD_*Mn*_ can significantly affect bacterioplankton community composition (Chen Z. J. et al., 2020). Sun et al. (2021) studied the composition of bacterioplankton in the upstream river of the DJR and reported that environmental parameters such as pH, TN, Cond, and NH_4_^+^–N significantly affected the composition of the bacterioplankton communities. The dry season at the DJR occurs from February to July, and the wet season occurs from August to January of the following year. The physical and chemical properties of the water body (temperature, nutrients, etc.) vary greatly during the different periods. DJR bacterioplankton may exhibit yearly and seasonal variation, but currently, there is no comprehensive comparative study on different years and seasons. The DJR area carries relatively large nitrogen and phosphorus loads, of which TN, the most important factor affecting water quality, significantly exceeds the standard (Shu et al., 2017; Chen et al., 2018). Bacterioplankton is a principal contributor to the N and P cycles. As noted by Zhang L. et al. (2021) in their study of bacterial communities in the MR-SNWD main water diversion canal, attention will be focusing on microbial communities that help remove N and P in the water body. Furthermore, microbial communities play an important role in ecological processes through direct and indirect interactions. Ecological network analysis technology has been applied in studies of bacterioplankton interactions (Kara et al., 2013; Chen Z. J. et al., 2020; Zhang J. et al., 2021). However, there is still little information regarding the interaction pattern among species of bacterioplankton in the DJR. The ecological network of the DJR bacterioplankton community was constructed using bioinformatics analysis, and key species were identified. These data are of great significance for analyzing and predicting the survival patterns of bacterioplankton communities in the DJR ecosystem. The mechanisms of biome assembly have always been one of the core issues of ecology, among which the neutral theory has become a research hotspot due to its simplicity and predictive ability and has been widely applied in terrestrial ecosystems such as forests and grasslands; neutral processes can play an important role in shaping biomes (Zhou and Ning, 2017; Zhang et al., 2018). The neutral theory has also been applied to bacterioplankton community assembly in aquatic ecosystems. Chen et al. (2019) showed that stochastic processes dominate microeukaryotic community assembly in the Tingjiang River. Zhang L. et al. (2021) showed that the bacterial communities in the MR-SNWD main canal were mainly shaped by a deterministic process and that stochasticity dominated microeukaryotic community assembly. Currently, there are no reports on bacterioplankton community assembly in the DJR, thus requiring attention.

Because the DJR is related to the safe operation of the MR-SNWD, studies on the composition, function and assembly of bacterioplankton communities are of great ecological significance. Based on the current research status for this topic, we pose the following research questions: (1) Are there yearly and seasonal variations in the composition and function (especially the N and P cycles) of the bacterioplankton communities in the DJR? (2) How do bacterioplankton communities interact with each other in the DJR, and do the ecological networks and core microbiome change between different years and seasons? (3) How does bacterioplankton community assembly occur in the DJR? Is the assembly determined by deterministic or stochastic processes?

## Materials and Methods

### Study Area and Sample Collection

**FIGURE 1:**
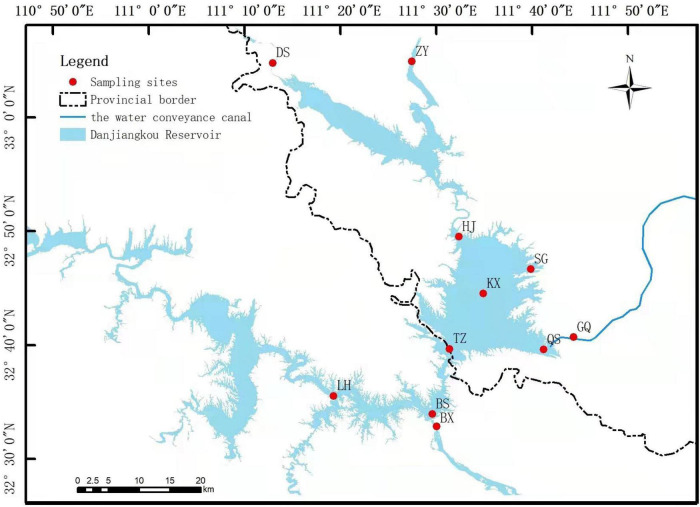
Location of study area and distribution of sampling sites.

According to geographic location, we set up 11 ecological sites in the DJR: Dashiqiao (DS), Zhangying (ZY), Heijizui (HJ), Kuxin (KX), Songgang (SG), Taizishan (TZ), Qushou (QS), Ganqu (GQ), Bashang (BS), Baxia (BX), Langhekou (LH) (Figure 1). The dry season at the DJR occurs from February to July, and the wet season occurs from August to January of the following year. We collected samples in the spring of 2018 (May, 2018S), the autumn of 2018 (October, 2018A), the spring of 2019 (May, 2019S), and the autumn of 2019 (October, 2019A). At each site, three replicate water samples were collected from surface water (0–50 cm). The water samples were processed for subsequent DNA extraction of bacterioplankton and physicochemical analysis of water quality. 0.22-μm filter paper was used to collect bacterioplankton by filter 1 L of water samples, and the filter were stored in a –80°C refrigerator.

Physicochemical variables were measured according to the environmental quality standard for surface water of China (GB3838-2002). Water temperature (T), pH and dissolved oxygen (DO) were measured *in situ* using the YSI 6920 (YSI Inc., Yellow Springs, Ohio, United States). Secchi depth (SD) was determined with a 30-cm-diameter Secchi disk. Water samples for chemical analysis were transported to the laboratory within 24 h, stored in a refrigerator at 4 °C, and analyzed within one week after sample collection. The permanganate index (COD_*Mn*_) was calculated using the potassium permanganate index method, and the chemical oxygen demand (COD) was measured by the potassium dichromate method. The total phosphorus (TP) was determined with acidified molybdate to form reduced phosphorus-molybdenum blue and measured spectrophotometrically. Total nitrogen (TN) was assayed *via* alkaline persulfate digestion and UV spectrophotometry, whereas ammonia nitrogen (NH_4_^+^–N) was measured using Nessler’s reagent spectrophotometric method. Chlorophyll a (Chl a) concentrations were estimated spectrophotometrically after extraction in 90% ethanol.

The trophic status of the Danjiangkou Reservoir area was assessed by measuring the parameters TN, TP, COD_*Mn*_, Chl a, and SD according to the improved Carlson’s trophic level index (TLI) (Wang et al., 2002; Chen Z. J. et al., 2020).

### DNA Extraction and Sequencing

DNA extraction from each filter was performed with the E.Z.N.A.^®^ Water DNA Kit (OMEGA, United States) following the instructions given by the supplier. The DNA samples were sent to Shanghai Majorbio Bio-Pharm Technology Co., Ltd. and sequenced (2 × 300) on an Illumina MiSeq platform. For the amplification of the 16S rRNA gene, the specific primers 338F (ACTCCTACGGGAGGCAGCA) and 806R (GGACTACHVGGGTWTCTAAT) were used for high-throughput pyrosequencing. Samples were amplified on a T100 thermal cycler (Bio-Rad Laboratories). After pyrosequencing, the raw data was filtered according to barcode and primer sequences using the software of Trimmomatic v0.39 and FLASH v1.2.11. Then the high-quality sequences were processed using the using QIIME 2 (Bolyen et al., 2019). Non-repeating sequences were extracted from the optimized sequences using UPARSE v7.0.1090 (DeSantis et al., 2006). The bacterial sequences were identified and clustered into OTUs (97% similarity) by using UCLUST (version 7.1^1^) method (Edgar, 2010). The high-thoughput sequencing data were deposited in the MG-RAST^2^ under accession number of mgp101864.

### Bioinformatic Analysis

We performed beta diversity analysis online using the free online platform of Majorbio Cloud Platform.^3^ The Unweighted pair-group method with arithmetic mean (UPGMA), partial least squares-discriminant analysis (PLS-DA), non-metric multidimensional scaling (NMDS), redundancy analysis (RDA), canonical correspondence analysis (CCA), variation partition analysis (VPA), correlation test and mantel test were conducted on this platform (Ren et al., 2022). Phylogenetic molecular ecological networks (pMENs) analysis was performed using the Molecular Ecological Network Analyses Pipeline (MENAP)^4^ (Zhou J. Z. et al., 2011; Deng et al., 2012). The metagenomes predicted by the Phylogenetic investigation of communities by reconstruction of unobserved states (PICRUSt2) algorithm were classified into clusters of orthologous groups (COGs) (Douglas et al., 2020). In this study, PICRUSt2 was used to explore the functional profiles of the bacterial communities according to the online protocol. Heat map were generated from the gene copy number of the functional genes using the TBtools software (Chen C. et al., 2020). To determine the potential importance of stochastic processes on community assembly, we used a neutral community model (NCM) to predict the relationship between OTU detection frequency and their relative abundance across the wider metacommunity. In this model, Nm is an estimate of dispersal between communities. The parameter Nm determines the correlation between occurrence frequency and regional relative abundance, with N describing the metacommunity size and m being the immigration rate. The parameter R^2^ represents the overall fit to the neutral model. Calculation of 95% confidence intervals around all fitting statistics was done by bootstrapping with 1000 bootstrap replicates.

## Results

### Physical and Chemical Properties of Water

Except for TN and COD_*Mn*_, the water quality of the DJR is generally good based on China’s *Environmental Quality Standards for Surface Water* (GB38382-2002) and, overall, meets the requirements of class I water standards. In 2018S, 2018A, 2019S and 2019A, the TN contents were high, with average contents of 1.75, 1.80, 1.63 and 1.96 mg/L, respectively, concentrations that were higher than the water quality standards for class IV surface water, with trends of higher concentrations in autumn than in spring (Supplementary Table 1). The COD_*Mn*_ was similar to TN, with average concentrations of 2.60, 2.72, 2.72, and 2.96 mg/L in 2018S, 2018A, 2019S, and 2019A, respectively, concentrations that met the class II surface water standards, with trends of higher concentration in autumn than in spring.

### Bacterioplankton Composition and Yearly and Seasonal Variations

**FIGURE 2:**
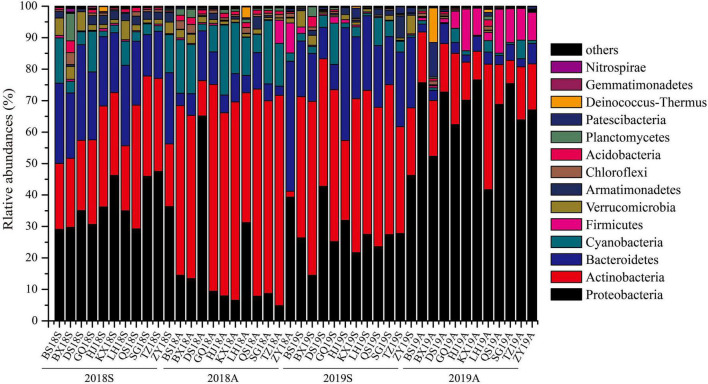
Relative abundance of bacterioplankton sequences at the phylum.

High-throughput sequencing (HTS) results indicated that the DJR bacterioplankton comprised the phyla Proteobacteria, Actinobacteria, Bacteroidetes, Cyanobacteria, Firmicutes, Verrucomicrobia, and Armatimonadetes, of which Proteobacteria, Actinobacteria and Bacteroidetes accounted for 71.78%∼96.98% of the total population (Figure 2). At the genus level, *CL500-29_marine_group*, *Acinetobacter*, *hgcI_clade*, *Limnohabitans*, *Cyanobium_PCC-6307*, *Flavobacterium*, *Brevundimonas*, *Sediminibacterium*, and *Exiguobacterium* accounted for 27.38% ∼ 96.60% of the bacterioplankton population (Supplementary Figure 1).

**FIGURE 3:**
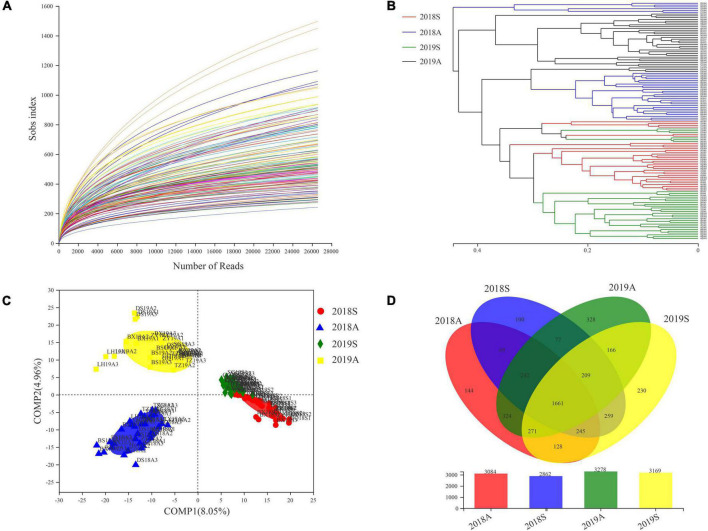
Dilution curve **(A)**, UPGMA-tree **(B)**, PLS-DA **(C)** and Venn diagram **(D)** base on pyrosequencing of bacterioplankton communities.

A dilution curve was used to evaluate the sequencing depth of the samples. The results indicated greater than 20,000 sample bands, with a dilution curve that tended to be flat (Figure 3A). UPGMA clustering tree analysis and PLS-DA were used to analyze the bacterioplankton community differences among different samples. In the UPGMA clustering tree, 2018S and 2019S were clustered in the lower part and separated from the 2018A and 2019A samples (Figure 3B). The PLS-DA analysis results were similar to the UPGMA clustering tree results. In the PLS-DA plot, 2018S and 2019S were distributed on the right side of the plot, and 2018A and 2019A were distributed on the left side of the plot, indicating that compared with different years, different seasons had a greater impact on the bacterioplankton communities and was the most important factor (Figure 3C). Additionally, comparing different seasons, the distances in the PLS-DA plots for 2018A and 2019A were greater than those for 2018S and 2019S, indicating that the bacterioplankton communities in the DJR varied in different seasons and that the variations in autumn were larger than those in spring. Samples from different seasons were clustered together in the UPGMA and PLS-DA plots, but the DS and ZY samples were poorly clustered. Based on the distribution of the sampling points, these two points were located on the tributaries of the reservoir, and thus, the bacterioplankton communities were different from those in the reservoir. Adonis and ANOISM were used to test the differences in the overall composition of the bacterioplankton communities (Supplementary Table 2). The results indicated that the differences in the composition of the bacterioplankton communities in different years and in different seasons were significant (*P* < 0.05). The number of OTUs in the samples at different time points was analyzed using a Venn diagram. The results indicated that the overall number of bacterial OTUs in the autumn was higher than that in the spring and higher in 2019 than in 2018 (Figure 3D).

### Influencing Factors of Bacterioplankton Communities

**FIGURE 4:**
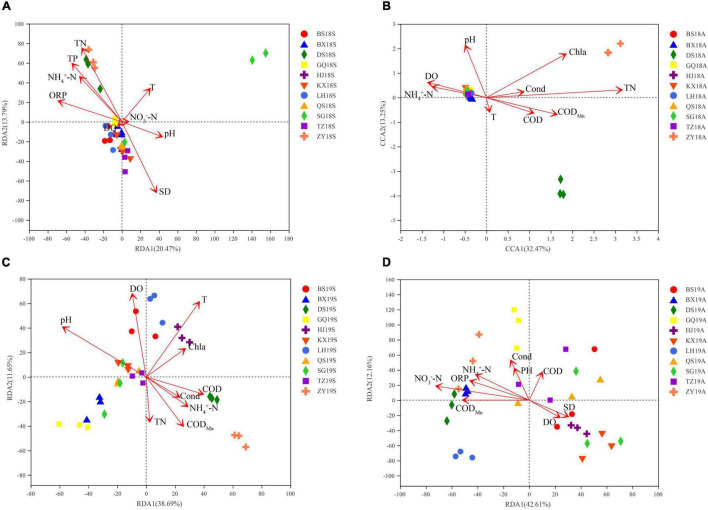
RDA, CCA of 2018S **(A)**, 2018A **(B)**, 2019S **(C)**, 2019A **(D)** bacterioplankton communities of and physico-chemical water quality parameters.

First, the environmental factors with a variance inflation factor (VIF) > 10 were screened and removed by VIF analysis, and the screened environmental factors were used for RDA or CCA. The effects of environmental factors in different years and seasons on the bacterioplankton communities were analyzed by RDA or CCA. ORP, TP, TN, NH_4_^+^-N and SD were the significant factors affecting the 2018S bacterioplankton communities (Figure 4A). pH, COD_*Mn*_, TN and Chla were the significant factors affecting the 2018A bacterioplankton communities (Figure 4B). T, pH, DO, COD, COD_*Mn*_, TN, NH_4_^+^-N and Chla were the significant factors affecting the 2019S bacterioplankton communities (Figure 4C). Cond, ORP, COD_*Mn*_, NH_4_^+^-N, and NO_3_^–^-N were the significant factors affecting the 2019A bacterioplankton communities (Figure 4D). The above results suggest that the composition and degree of impact of the significant environmental factors vary between different years and seasons and that the relationship between the DJR bacterioplankton communities and environmental factors exhibits annual and seasonal variations. Excessive N and P nutrients in the DJR are the most important factors affecting water quality in the reservoir. N and P nutrients are also the main factors affecting the bacterial communities. The effects of N and P nutrients (TN, NH_4_^+^-N, NO_3_^–^-N, TP) and other environmental factors on the changes in bacterial community composition were analyzed by VPA. N and P nutrients (TN, NH_4_^+^-N, NO_3_^–^-N, TP) explained 16.12%, 10.59%, 32.46%, and 24.93% of the variation in bacterial community composition in 2018S, 2018A, 2019S, and 2019A, respectively, indicating a strong interaction of N and P nutrients in shaping the microbial communities in the reservoir.

### Bacterioplankton Functions

**FIGURE 5:**
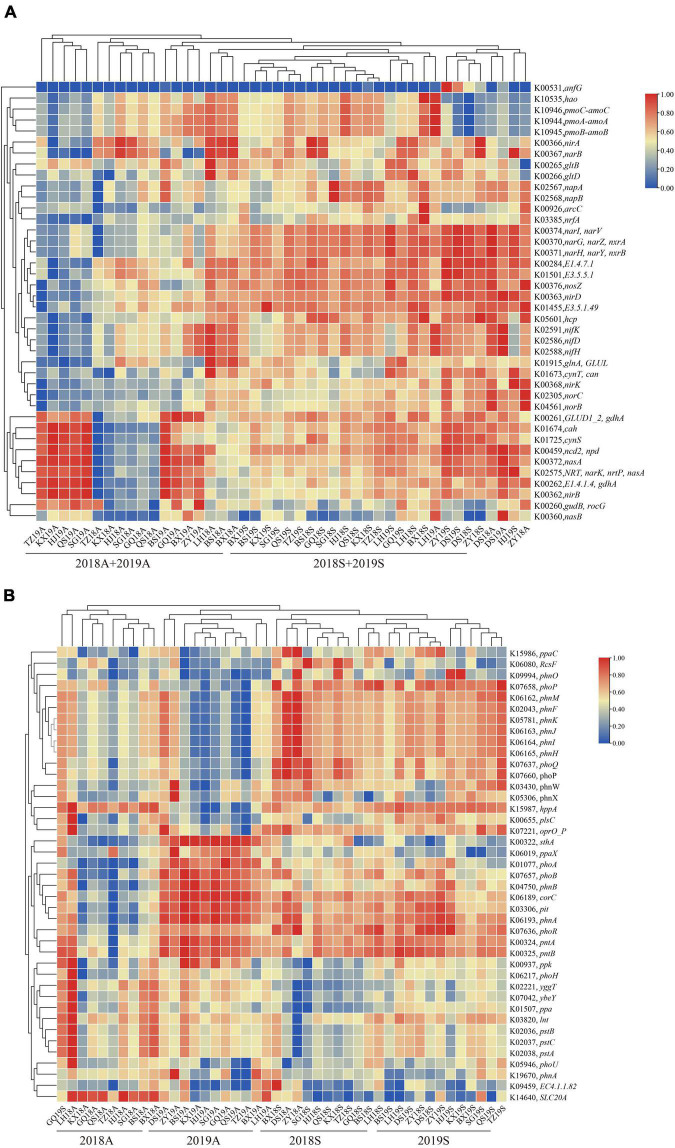
Heatmap showing the hierarchical clustering of the predicted functional genes related to the N **(A)** and P **(B)** cycles in bacterioplankton sample.

To assess the functions of bacterioplankton at different site in the DJR, PICRUSt2 software was used to perform microbiota predictions and analyses. The prediction results, based on the COG database, included a total of 24 functional gene families, of which six functional gene families, such as amino acid transport and metabolism and translation, Translation, ribosomal structure and biogenesis, Energy production and conversion, Cell wall/membrane/envelope biogenesis, Inorganic ion transport and metabolism, Carbohydrate transport and metabolism were the main functional gene families, accounting for 40.29%–45.76%. We analyzed the functional genes related to the N and P cycles in bacterioplankton samples from the DJR for different years and seasons. The results indicated that such genes were predominantly involved in nitrogen fixation (K02588 *nifH*), nitrification (K10535 *hao*), denitrification (K00368 *nirK*, K04561 *norB*, and K00376 *nosZ*), nitrogen assimilation reduction and dissimilarity reduction (K02575 *nasA*, K00367 *narB*, K02567 *napA*, K00366 *nirA*, K00362 *nirB*, and K03385 *nrfA*) and other related nitrogen cycle function genes. The results of cluster analysis of the copy number of nitrogen cycle genes indicated two separate groups in spring and autumn, indicating that season was the most important factor affecting the bacterioplankton N cycle (Figure 5A). The 2018 and 2019 samples were separated from each other in different seasons, indicating that year was also an important influencing factor. The predicted key genes of the P cycle were K00655 *plsC*, K01507 *ppa*, K02036 *pstB*, K02037 *pstC*, K02038 *pstA*, K00324 *pntA*, K06217 *phoH*, K03820 *lnt*, and K07636 *phoR.* The results of the gene copy number cluster analysis were similar to those for the N cycle, i.e., season and year were the main influencing factors (Figure 5B).

### Ecological Network Analysis

**FIGURE 6:**
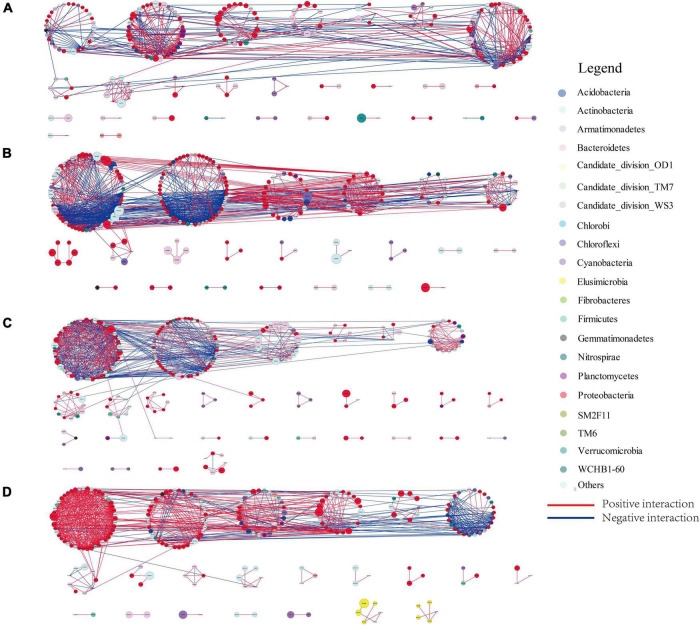
Overview of the 2018S **(A)**, 2018A **(B)**, 2019S **(C)**, 2019A **(D)** bacterioplankton networks. Each node represents an OTU. The colored circles indicate the OTUs affiliated with particular phyla (color code on the right).

The relative abundance of bacterioplankton was used to construct a bacterioplankton molecular ecological network for the DJR bacterioplankton in different years and seasons based on the RMT method (Figure 6). Based on the analysis of the network properties, the number of nodes in the 2019 sample was higher than that in the 2018 sample, and it was higher in the spring than in the autumn. The main trend for the total number of links was that the number was lower in spring than in autumn. Additionally, the average clustering coefficient and average connectivity of the molecular ecological network for the spring samples were lower than those for the autumn samples, and the number of modules were higher for the spring samples than for the autumn samples.

**FIGURE 7:**
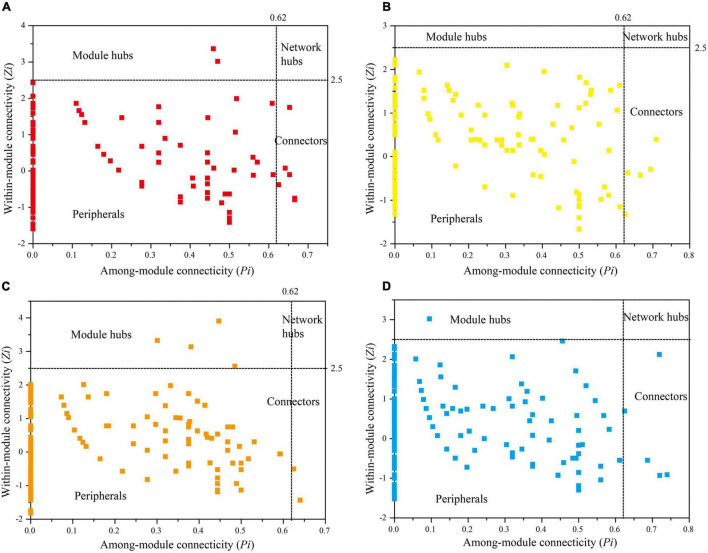
*Zi*-*Pi* plot of 2018S **(A)**, 2018A **(B)**, 2019S **(C)**, 2019A **(D)** bacterioplankton networks.

In addition, molecular ecological networks have been widely used in the identification of core microbiomes. Nodes with *Zi* ≥ 2.5 or *Pi* ≥ 0.62 were defined as core microbiomes. The number and composition of core microbiomes in the molecular ecological network of bacterioplankton in different years and seasons in the DJR were different. There were 8 core microbiomes (OTU number) for the 2018S sample: OTU2563 and OTU3223 of Actinobacteria, OTU1049, OTU3534, and OTU3798 of Bacteroidetes, OTU2938 of Cyanobacteria, OTU2425 of Gemmatimonadetes, and OTU1780 of Proteobacteria (Figure 7A). There were 5 core microbiomes (OTU number) for the 2018A sample: OTU1765 of Actinobacteria, OTU89 of Bacteroidetes, OTU12 of Proteobacteria, OTU3233 of Verrucomicrobia (Figure 7B). There were 6 core microbiomes (OTU number) for the 2019S sample: OTU3748, OTU4243 and OTU3770 of Bacteroidetes, OTU1931, OTU2817 and OTU3044 of Proteobacteria (Figure 7C). There were 6 core microbiomes (OTU number) for the 2019A sample: OTU2082, OTU3717 of Actinobacteria, OTU2641 of Firmicutes, OTU1976 of Patescibacteria, OTU2034, OTU960 of Proteobacteria (Figure 7D). The OTU classification information for these key bacteria is provided in Supplementary Table 3. Spearman correlation was used to analyze the relationship between environmental factors and key bacteria. The results showed that T, Cond, COD_*Mn*_, TP, NH_4_^+^-N, NO_3_^–^-N and SD were the main factors affecting core microbiomes.

### Community Assembly

**FIGURE 8:**
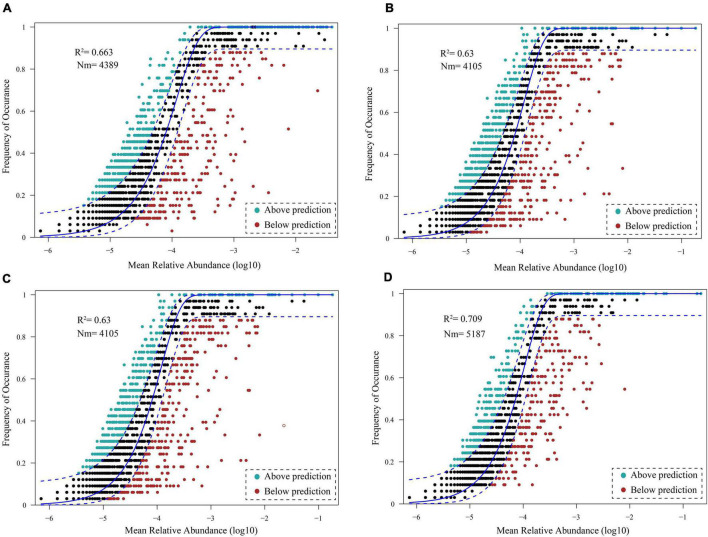
Fit of the neutral community model (NCM) of 2018S **(A)**, 2018A **(B)**, 2019S **(C)**, 2019A **(D)** bacterioplankton community assembly.

Neutral model analysis of the bacterioplankton communities in the DJR showed that the neutral model explained 66.3%, 63.0%, 63.0%, and 70.9% of the bacterioplankton community variations in 2018S (Figure 8A), 2018A (Figure 8B), 2019S (Figure 8C), and 2019A (Figure 8D), respectively, and that stochastic processes played a leading role.

## Discussion

### The Composition of and Yearly and Seasonal Variation in the Bacterioplankton Communities in the Danjiangkou Reservoir

High-throughput sequencing (HTS) has been widely used in the study of bacterial community composition in aquatic ecosystems, giving people the opportunity to conduct more in-depth and comprehensive studies on the structure and function of bacterial communities in freshwater (Grossart, 2010). Liu et al. (2015) analyzed the bacterioplankton composition of 42 lakes and reservoirs in China. The results indicated that Actinobacteria, Bacteroidetes, and Cyanobacteria were the dominant populations. Sun et al. (2021) analyzed Han River bacterioplankton upstream of the DJR. The results showed that the dominant genera were *Flavobacterium*, *Planktophila*, and *Siphonobacter*. Our assessment of the bacterioplankton communities in the DJR showed that they were mainly composed of bacterial phyla common in water bodies, such as Proteobacteria, Actinobacteria, and Bacteroidetes. At the genus level, the communities were mainly composed of *CL500-29_marine_group*, *Acinetobacter*, *hgcI_clade*, and *Limnohabitans*. The community composition was similar to the composition of DJR bacterioplankton in May 2016 but differed from the composition of bacterioplankton in the main water diversion canal in the Han River, upstream of the DJR (Luo et al., 2019; Chen Z. J. et al., 2020; Zhang L. et al., 2021).

Many studies have shown that bacterioplankton exhibit temporal and spatial variation characteristics under the influence of physicochemical properties such as T, pH, nutrients, and DO in the water body and hydrological factors such as flow velocity, flow rate, and water level (Pearce, 2005; Qian-Qian et al., 2021). The dry season at the DJR occurs from February to July, and the wet season occurs from August to January of the following year. The water level of, nutrients in, and temperature of the reservoir vary greatly during different periods (Dong et al., 2020). Based on our analysis, the physicochemical properties of the water body were different in different periods, and T, TN, and COD_*Mn*_ exhibited trends of being higher in the autumn than in the spring (Supplementary Table 1). UPGMA clustering and PLS-DA of bacterioplankton community composition revealed that bacterioplankton communities were significantly different in different seasons and years and that the influence of season was the most important factor. This finding is consistent with the results reported by Luo et al. (2019), who showed that the seasonal variation in the composition of the bacterioplankton communities in the main canal was greater than that of the spatial distribution variation.

Seasonal variation in bacterioplankton can be reflected not only by community composition but also through the interaction and composition and function of core microbiomes. Jiao et al. (2020) conducted a network analysis of planktonic bacterial communities and showed seasonal variations in bacterioplankton network attributes. Molecular ecological network analysis of DJR bacterioplankton showed that network attributes such as nodes, links, average clustering coefficient, average connectivity, and modules all exhibited seasonal variations. These network attributes are used to indicate the size, complexity, efficiency of transferring matter, energy and information between species, and sensitivity to the external environment of the network (Zhou J. et al., 2011; Chen et al., 2021). The links, average clustering coefficient, and average connectivity for the autumn samples were all higher than those for the spring samples, indicating a more complex network association and higher susceptibility to interference from the external environment. Similarly, an analysis of the bacterioplankton network for the four seasons at Lake Taihu indicated that the network in autumn was the most complex and the network in spring was the simplest (Lin et al., 2019). The reason may be due to the massive reproduction of phytoplankton in autumn. The large amount of soluble organic matter, including hydrocarbons and organic acids, produced by phytoplankton photosynthesis provides abundant nutrients for bacteria, allowing bacterioplankton networks to be more complex. Another important function of network analysis is the identification of core microbiomes, which play important roles in the stabilization of ecological service functions. The results showed that the number of core microbes in 2018S was higher than that in 2018A. The number of core microbiomes in 2019 was the same, but the composition of core microbiomes was significantly different. The relative abundance of bacterial communities to which some key nodes belong to in the Danjiangkou bacterial network was relatively low (< 1%), indicating that low-abundance bacteria in the water body played an important role in bacterial network assembly and suggesting that further attention should be paid to the role of low-abundance bacteria (Liu et al., 2015; Peter et al., 2018). The molecular network analysis used to investigate the links between numerous taxa, however, these links are often difficult to provide any evidence of such interactions using biochemical or other standard microbiological tests referring to living microbes. Thus, it is required follow up experimental validation to confirm true bacterial interactions in Danjiangkou Reservoir.

### N and P Nutrients Are the Main Factors Driving the Communities and Function of Bacterioplankton in the Danjiangkou Reservoir

The DJR is an important drinking water source in China, and water quality is always stable at classes II and above. A claim supported by the monitoring results from this experiment (Supplementary Table 1). Due to the impact of non-point source pollution, such as agricultural production around the DJR and the input of main and tributary streams, such as the Hanjiang River and the Danjiang River, the excessive TN content in the DJR has become a major threat to water quality in the water body. Dong et al. (2020) conducted TN analysis of the DJR for 2016-2020 and showed that the TN content ranged from 0.928 to 1.020 mg/L. The results of this experiment showed that the TN content in the DJR exceeded 1.00 mg/L, with the highest content being 2.862 mg/L, meeting the standard for class IV surface water and showing a trend of higher concentrations in autumn than in spring. An explanation for thus finding is that autumn is the wet season, and N in the soil of the upstream basin enters the water body with rain. As the main factor in freshwater ecosystems, N and P nutrients significantly affect the composition of bacterioplankton communities (Kisand et al., 2001; Maria Montserrat et al., 2002). An RDA of the bacterioplankton communities and environmental factors in the DJR in May 2016 showed that TN was an important factor affecting the distribution of bacterioplankton (Chen Z. J. et al., 2020). In this study, RDA and CCA indicated that N and P nutrients significantly affected the composition of the bacterioplankton communities, and subsequent VPA analysis indicated that N and P nutrients were the most important factors affecting the bacterioplankton communities. Previous long-term monitoring of the DJR showed that it was in a mesotrophic state and that N and P contents in the DJR were lower than those in other lakes in China (Chen Z. J. et al., 2020). Many previous studies have shown that in lakes the nutrient concentration may be the limiting factor for bacterial growth and is the main factor affecting the composition of bacterioplankton communities (Vrede, 2005). Spearman correlation analysis showed that NH_4_^+^-N, NO_3_^–^-N, TP and other nutrients were also the main factors affecting core microbiomes, indicating that they can also affect bacterioplankton interactions.

Microbes are also the main driving force of the N and P cycles in the DJR. Dang et al. (2021) found nitrogen-cycle bacteria such as *Rhodoferax*, *Polaromonas*, *Limnohabitans*, *Pararheinheimera*, *Desulfobulbus*, and *Pseudopelobacter* as well as 51 nitrogen functional genes in the DJR. Studies have shown that PICRUSt can accurately predict the presence and abundance of functional genes (Hartman et al., 2017; LeBrun and Kang, 2018; Ribeiro et al., 2018). We analyzed the functional genes related to the N and P cycles in the DJR in different years and seasons. PICRUSt2 predicted 40 N functional genes and 41 P functional genes, findings that were consistent with the functional genes of bacterioplankton for N cycling in Pearl River Estuary predicted by Zhu et al. (2018) and the functional genes of bacterioplankton for P cycling in Poyang Lake predicted by Ren et al. (2019). These results indicated that the bacterioplankton communities in the DJR had abundant N and P cycle functional genes. Cluster analysis of N cycle gene copy number indicated that similar to the composition of the bacterial communities, season was the most important factor affecting the bacterioplankton N cycle. The above results indicate that N and P nutrients are the main factors driving the communities and function of bacterioplankton in the DJR. Similar to other lakes and reservoirs, T, pH, COD, COD_*Mn*,_ Cond, and Chla are also important factors affecting the composition of the bacterioplankton communities in the DJR (Liu et al., 2015; Chen Z. J. et al., 2020; Dong et al., 2020; Qian-Qian et al., 2021; Sun et al., 2021).

### Both Stochastic Processes and Deterministic Processes Dominate Bacterioplankton Community Assembly in the Danjiangkou Reservoir

Currently, there is no study on bacterioplankton community assembly in the DJR. Relevant studies are not only conducive to predicting variations in community composition but also play a potential role in bacterioplankton ecological function and diversity protection (Nemergut et al., 2013). Niche theory and neutral theory are the main models that explain the formation and maintenance of biodiversity, that is, the community assembly. The niche theory proposes that biological communities are regulated by environmental selection and biological interaction, which are deterministic processes; the neutral theory proposes that stochastic processes, including the birth, death, migration, and diffusion of species, shape biological communities (Zhou and Ning, 2017; Zhang et al., 2018; Chen et al., 2019). Researchers have tried to strengthen the understanding of the influence of stochastic processes on bacterioplankton community assembly based on the neutral theory. In this study, Environmental factors such as T, pH, DO, COD, COD_*Mn*_, TN, NH_4_^+^-N and Chla were significantly related to variations in the bacterioplankton community. They demonstrated that environmental factors have impact on bacterial community assembly. But, the high proportion of unexplained variation in bacterioplankton communities indicated the potential importance of neutral or stochastic processes for community assembly. Our analysis of the neutral model of bacterioplankton communities in the DJR showed that the neutral model explained 66.3%, 63.0%, 63.0%, and 70.9% of the bacterioplankton community variation in 2018S, 2018A, 2019S, and 2019A, respectively. The neutral community model fitted well for the bacterioplankton community with a moderate fitted value (R^2^ = 0.630∼0.709). The fitted value indicated that stochastic process played only a moderate role in the community assembly process by comparing with other studies (Chen et al., 2019; Zhang Z. F. et al., 2021; Zhang et al., 2022). Due to the heightening of the dam and the increase in the water level, the aquatic ecosystem in the DJR is in a process of reconstruction. Additionally, due to the demand for water storage and water transfer, the hydrological and water quality physicochemical properties of the reservoir during the dry season and the wet season vary greatly each year, potentially affecting neutral (dispersal-related) process in the DJR and making it mainly a stochastic process.

## Conclusion

The water quality in the DJR is important because it is associated with the safety of drinking water for hundreds of millions of people along the MR-SNWD in China. In view of the important role of bacterioplankton in aquatic ecosystems, the study of bacterioplankton community composition, function and community assembly has important ecological and economic significance. We analyzed the bacterioplankton community composition and distribution characteristics in different years and seasons and found that bacterioplankton had annual and seasonal variations. Additionally, the main factors affecting the bacterial communities were analyzed through RDA and CCA, and it was found that N and P nutrients were the main driving factors. Subsequently, the function of bacterioplankton was predicted by PICRUSt2, and it was found that the N and P cycle functions of bacterioplankton had significant seasonality. In addition, ecological network analysis revealed that the links, average clustering coefficient, and average connectivity of the autumn samples were all higher than those of the spring samples, indicating that the network was more complex and more susceptible to interference from the external environment. The analysis of the neutral model showed that stochastic processes dominated bacterioplankton community assembly in the DJR. This study systematically studied the composition, function, interaction, and assembly of the bacterioplankton communities in the DJR as well as the influencing factors, providing a reference for the protection of water quality and the ecological functions of DJR bacterioplankton.

## Data Availability Statement

The datasets presented in this study can be found in online repositories. The names of the repository/repositories and accession number(s) can be found below: MG-RAST metagenomics analysis server, accession number: mgp101864 (https://www.mg-rast.org/linkin.cgi?project=mgp101864).

## Author Contributions

Z-JC: conceptualization, investigation, and writing – original draft. Y-QL, L-AL, and B-HZ: investigation. Y-YL: conceptualization, funding acquisition, and writing – review and editing. M-FJ: methodology and investigation. BL: conceptualization and writing – review and editing. X-MH: conceptualization, methodology, and writing – original draft. All authors contributed to the article and approved the submitted version.

## Conflict of Interest

The authors declare that the research was conducted in the absence of any commercial or financial relationships that could be construed as a potential conflict of interest. The reviewer LY declared a shared affiliation with the authors Z-JC, Y-QL, Y-YL, L-AL, B-HZ, and M-FJ to the handling editor at the time of review.

## Publisher’s Note

All claims expressed in this article are solely those of the authors and do not necessarily represent those of their affiliated organizations, or those of the publisher, the editors and the reviewers. Any product that may be evaluated in this article, or claim that may be made by its manufacturer, is not guaranteed or endorsed by the publisher.

## Footnotes

1
http://drive5.com/uparse/

2
http://www.mg-rast.org/

3
www.majorbio.com

4
http://ieg4.rccc.ou.edu/mena/login.cgi
