# Supplementary material for: The Seasonal Patterns, Ecological Function and Assembly Processes of Bacterioplankton Communities in the Danjiangkou Reservoir, China
Source: Front Microbiol. 2022 Jun 15;13:884765. doi: 10.3389/fmicb.2022.884765 (PMC9240478; doi:10.3389/fmicb.2022.884765)
Supplement: Supplementary file 1 [file Data_Sheet_1.docx]

**Supporting Materials**

**The seasonal patterns, ecological function and assembly processes of bacterioplankton communities in the Danjiangkou Reservoir, China**

Zhao-Jin Chen ^1^, Yong-Qi Liu^1^, Yu-Ying Li^1,^*, Li-An Ling^1^, Bao-Hai Zheng^1^, Ming-Fei Ji^1^, B. Larry Li^2^, Xue-Mei Han^3,^*

Corresponding author.

E-mail: lyying200508@163.com (Y-Y, Li); hanxuemei916@163.com (X-M, Han)

^1^ International Joint Laboratory of Watershed Ecological Security and Collaborative Innovation Center of Water Security for Water Source Region of Middle Route Project of South-North Water Diversion in Henan Province, School of Water Resource and Environmental Engineering, Nanyang Normal University, Nanyang 473061, China

^2^ Ecological Complexity and Modelling Laboratory, Department of Botany and Plant Sciences, University of California, Riverside, CA 92521-0124, USA

^3^ Ministry of Education Key Laboratory for Ecology of Tropical Islands, College of Life Sciences, Hainan Normal University, Haikou 571158, China

Table 1. Main physicochemical characteristics and trophic level index (TLI) of water samples (means ± S.E.).

| Sample | T (℃) | pH | DO (mg/L) | Cond (ms/cm) | ORP (mv) | COD (mg/L) | CODMn (mg/L) | TP (mg/L) | TN (mg/L) | NH_4_^+^-N  (mg/L) | NO_3_^-^-N(mg/L) | Chla (mg/m^3^) | SD (m) | TSI |
| --- | --- | --- | --- | --- | --- | --- | --- | --- | --- | --- | --- | --- | --- | --- |
| ZY18S | 20.4±0.08 | 7.67±0.05 | 6.27±0.21 | 479.67±7.76 | 264.67±9.03 | 12.33±0.21 | 2.94±0.04 | 0.075±0.002 | 2.68±0.01 | 0.37±0.01 | 1.08±0 | 7.39±0.04 | 1.10±0.08 | 44.13±2.71 |
| DS18S | 19.63±0.05 | 8.13±0.12 | 5.27±0.12 | 467.33±4.64 | 268.67±5.79 | 11.37±0.09 | 3.03±0.02 | 0.037±0.001 | 3.64±0 | 0.07±0.04 | 1.06±0 | 2.12±0.07 | 1.03±0.05 | 36.15±2.08 |
| QS18S | 17.97±0.05 | 8.30±0.08 | 5.50±0.08 | 263.33±2.87 | 206±3.56 | 10.57±0.24 | 2.05±0.01 | 0.010±0.001 | 1.59±0 | 0.08±0 | 1.35±0 | 1.17±0.03 | 4.10±0.14 | 31.93±0.22 |
| GQ18S | 20.43±0.05 | 8.4±0.08 | 4.73±0.05 | 262.33±4.78 | 179.33±1.7 | 9.9±0.08 | 2.16±0.02 | 0.031±0.002 | 1.26±0 | 0.08±0 | 1.04±0 | 1.22±0.02 | 4.80±0.16 | 32.35±0.06 |
| SG18S | 21.57±0.05 | 8.47±0.05 | 5.93±0.12 | 271.67±4.5 | 170.00±0.82 | 8.57±0.25 | 2.02±0.01 | 0.009±0 | 1.46±0 | 0.04±0 | 1.10±0 | 0.87±0.01 | 4.53±0.05 | 29.00±0.29 |
| KX18S | 22.1±0.08 | 8.30±0.08 | 5.33±0.12 | 267.33±1.25 | 243±2.16 | 12.43±0.24 | 2.34±0.03 | 0.009±0 | 1.58±0 | 0.09±0 | 0.98±0 | 0.36±0 | 5.87±0.09 | 20.01±0.27 |
| HJ18S | 20.07±0.05 | 8.23±0.05 | 8.03±0.12 | 274.67±2.62 | 254±2.94 | 11.57±0.21 | 2.71±0.02 | 0.015±0.002 | 1.07±0 | 0.09±0 | 1.04±0 | 0.58±0.03 | 4.00±0 | 24.66±0.13 |
| TZ18S | 18.2±0.08 | 8.2±0.08 | 4.67±0.17 | 283.33±2.05 | 204.67±3.4 | 11.17±0.19 | 1.84±0.04 | 0.009±0 | 1.09±0 | 0.09±0 | 0.95±0 | 1.22±0.02 | 5.07±0.09 | 31.63±1.09 |
| BS18S | 15.97±0.05 | 7.4±0.08 | 7.77±0.09 | 255.33±2.49 | 264.33±3.09 | 14.7±0.29 | 3.51±0 | 0.03±0.001 | 1.74±0 | 0.10±0 | 1.11±0 | 0.74±0.02 | 4.20±0.14 | 24.78±1.67 |
| LH18S | 17.7±0.08 | 8.30±0.08 | 5.30±0.08 | 254.33±0.94 | 220±0.82 | 14.6±0.08 | 2.93±0.01 | 0.034±0.001 | 1.58±0 | 0.14±0 | 0.97±0 | 1.32±0.02 | 5.77±0.21 | 33.2±0.21 |
| BX18S | 14.4±0.08 | 7.73±0.17 | 7.03±0.12 | 236.33±2.49 | 204±3.27 | 13.57±0.25 | 3.12±0.03 | 0.05±0.001 | 1.51±0 | 0.1±0 | 1.37±0 | 0.39±0.02 | 2.4±0.14 | 23.19±5 |
| ZY18A | 24.57±0.05 | 8.43±0.12 | 7.6±0.08 | 223.33±2.05 | 198.33±0.94 | 13.7±0.14 | 3.19±0.02 | 0.062±0 | 3.13±0 | 0.02±0 | 3.02±0 | 4.62±0.24 | 1.00±0 | 45.57±0.51 |
| DS18A | 25.6±0.22 | 7.8±0.08 | 7.53±0.05 | 200.33±1.7 | 265±0.82 | 14.07±0.12 | 3.16±0.03 | 0.03±0.001 | 2.25±0 | 0.02±0 | 2.20±0 | 1.24±0.03 | 1.07±0.09 | 32.18±0.07 |
| QS18A | 24.2±0.08 | 8.13±0.12 | 8.87±0.05 | 228.67±0.47 | 208.33±2.05 | 14.27±0.17 | 2.81±0.02 | 0.007±0.001 | 1.85±0 | 0.03±0 | 1.34±0 | 1.14±0.01 | 4.17±0.12 | 31.39±0.18 |
| GQ18A | 22.23±0.05 | 8.67±0.09 | 7.63±0.05 | 211.67±0.47 | 165.67±2.62 | 9.53±0.05 | 1.67±0.02 | 0.013±0 | 1.17±0 | 0.06±0 | 0.92±0 | 1.24±0.03 | 4.93±0.09 | 31.67±1.11 |
| SG18A | 27.47±0.05 | 8.17±0.09 | 7.73±0.05 | 171±1.63 | 300.33±3.3 | 14±0.08 | 2.69±0.02 | 0.014±0.001 | 1.73±0 | 0.07±0 | 1.19±0.01 | 1.49±0.03 | 4.00±0 | 32.34±1.93 |
| KX18A | 26.4±0.08 | 8.23±0.05 | 7.87±0.05 | 147±1.41 | 299.33±4.03 | 14.17±0.09 | 2.23±0.01 | 0.009±0 | 1.80±0 | 0.06±0 | 1.22±0.01 | 0.98±0.05 | 5.10±0.14 | 30.3±0.47 |
| HJ18A | 25.33±0.05 | 8.27±0.09 | 8.33±0.05 | 107.33±3.09 | 285±2.16 | 11.37±0.09 | 2.98±0.02 | 0.011±0 | 1.82±0 | 0.11±0 | 1.39±0 | 1.52±0.02 | 3.53±0.05 | 34.45±0.16 |
| TZ18A | 26.23±0.05 | 7.97±0.25 | 9.43±0.12 | 201.67±0.47 | 322±5.1 | 11.43±0.12 | 2.68±0.02 | 0.01±0 | 1.67±0 | 0.01±0 | 1.25±0 | 2.14±0.02 | 3.47±0.12 | 35.03±2.43 |
| BS18A | 25.83±0.05 | 8.27±0.05 | 7.8±0.08 | 222±0.82 | 280.67±1.25 | 12.27±0.12 | 2.74±0.01 | 0.018±0.001 | 1.51±0 | 0.04±0 | 1.08±0 | 2.01±0.02 | 5.00±0 | 36.57±0.98 |
| LH18A | 24.53±0.17 | 8.23±0.12 | 8.07±0.05 | 220.33±1.25 | 241.67±1.7 | 13.33±0.12 | 2.94±0.04 | 0.017±0 | 1.35±0 | 0.04±0 | 1.2±0 | 3.41±0.12 | 4.33±0.25 | 41.71±0.59 |
| BX18A | 22.07±0.05 | 8.57±0.05 | 7.77±0.12 | 204±0.82 | 165.67±2.62 | 13.17±0.05 | 2.85±0.01 | 0.021±0.002 | 1.59±0 | 0.02±0 | 1.02±0 | 0.35±0.02 | 2.5±0.24 | 19.78±0.41 |
| ZY19S | 15.77±0.05 | 7.53±0.05 | 8.13±0.02 | 315±1.41 | 242.33±2.49 | 15.1±0.37 | 4.39±0.04 | 0.016±0.001 | 1.44±0 | 0.07±0 | 1.04±0 | 1.7±0.02 | 2.57±0.05 | 35.6±0.21 |
| DS19S | 14.97±0.09 | 7.83±0.09 | 8.35±0.03 | 227.67±1.25 | 235±1.41 | 11.9±0.16 | 2.15±0.01 | 0.06±0.001 | 2.86±0 | 0.54±0 | 1.45±0 | 3.31±0.04 | 1.23±0.05 | 39.9±3.04 |
| QS19S | 13.7±0.08 | 8.2±0.08 | 8.86±0.07 | 226.33±2.49 | 242±51.62 | 11.43±0.12 | 2.77±0 | 0.018±0 | 1.27±0 | 0.03±0 | 0.88±0 | 0.72±0.02 | 4.63±0.12 | 26.37±0.93 |
| GQ19S | 12.07±0.05 | 8.43±0.05 | 8.23±0.02 | 280±2.16 | 215±5.35 | 11.3±0.16 | 2.99±0.07 | 0.005±0 | 1.78±0 | 0.11±0 | 1.47±0.38 | 0.88±0.02 | 5.53±0.05 | 27.26±2.34 |
| SG19S | 14.37±0.09 | 8.27±0.05 | 8.71±0.03 | 267.33±0.47 | 238±3.74 | 12.27±0.05 | 2.05±0.01 | 0.017±0.001 | 1.22±0 | 0.03±0 | 0.91±0 | 1.1±0.06 | 3.97±0.05 | 31.25±0.31 |
| KX19S | 15.1±0.08 | 8.13±0.05 | 8.72±0.02 | 263±1.63 | 186.67±2.05 | 11.3±0.22 | 1.81±0 | 0.015±0 | 1.3±0 | 0.03±0 | 0.91±0 | 1.21±0.04 | 6.13±0.09 | 32.26±0.41 |
| HJ19S | 17.53±0.05 | 8.3±0.08 | 9.21±0.08 | 333±3.56 | 138±1.63 | 12.53±0.17 | 2.68±0.01 | 0.019±0.002 | 1.38±0 | 0.03±0 | 0.92±0 | 1.31±0.02 | 5.60±0.14 | 33.13±0.16 |
| TZ19S | 13.77±0.05 | 8.23±0.05 | 8.56±0.04 | 278.33±1.7 | 264.67±2.87 | 10.77±0.05 | 3.23±0.02 | 0.017±0.001 | 1.64±0 | 0.05±0 | 0.93±0 | 0.64±0.02 | 6.1±0.14 | 26.03±0.36 |
| BS19S | 18.77±0.12 | 8.53±0.05 | 8.99±0.03 | 230.33±1.25 | 198.67±2.05 | 11.47±0.12 | 2.89±0.01 | 0.017±0.001 | 1.15±0 | 0.03±0 | 0.64±0 | 5.45±0.05 | 3.57±0.05 | 45.82±1.15 |
| LH19S | 19.2±0.08 | 8.33±0.05 | 8.93±0.02 | 244.67±2.62 | 225.33±3.09 | 13.57±0.05 | 2.08±0.03 | 0.017±0.001 | 1.21±0 | 0.04±0 | 0.78±0 | 1.14±0.02 | 7.73±0.19 | 31.44±0.41 |
| BX19S | 13.07±0.05 | 8.27±0.12 | 8.57±0.05 | 252.33±0.94 | 186.67±2.62 | 13.4±0.08 | 2.92±0.04 | 0.011±0.001 | 2.84±0 | 0.12±0 | 2.28±0.01 | 0.37±0.02 | 2.57±0.05 | 20.29±0.19 |
| ZY19A | 23.77±0.12 | 8.4±0.01 | 5.4±0.07 | 387.67±6.24 | 260.67±1.25 | 9.07±0.04 | 3.19±0.01 | 0.035±0.002 | 1.75±0.04 | 0.06±0 | 1.61±0.01 | 2.06±0.03 | 2.17±0.12 | 37.32±0.18 |
| DS19A | 19.8±0.08 | 8.49±0.01 | 8.06±0.01 | 301.67±3.3 | 222.67±2.62 | 12.89±0.24 | 4.3±0.01 | 0.033±0.002 | 2.83±0.03 | 0.25±0.01 | 2.39±0.03 | 2.51±0.05 | 1.53±0.05 | 39.47±0.23 |
| QS19A | 24.97±0.12 | 8.57±0.02 | 6.91±0.04 | 245±2.16 | 199±1.63 | 13.22±0.27 | 2.44±0.02 | 0.013±0.003 | 1.44±0.01 | 0.05±0 | 1.25±0.04 | 0.41±0 | 4.27±0.21 | 21.27±1.04 |
| GQ19A | 24.43±0.12 | 8.65±0.01 | 7.1±0.03 | 246±2.16 | 197.67±1.25 | 10.72±0.18 | 2±0.01 | 0.029±0.001 | 1.82±0.01 | 0.13±0 | 0.99±0.01 | 0.87±0.02 | 5.1±0.08 | 27.48±2.48 |
| SG19A | 25.43±0.12 | 8.58±0.01 | 7.01±0.04 | 254±2.94 | 194.33±0.94 | 11.42±0.07 | 3±0.02 | 0.01±0.001 | 1.27±0.05 | 0.04±0 | 1.08±0 | 0.4±0.02 | 3.27±0.05 | 24.11±4.45 |
| KX19A | 25.73±0.05 | 8.55±0 | 7.13±0.02 | 245.67±1.25 | 218.67±1.25 | 7.53±0.03 | 2.23±0.02 | 0.015±0.001 | 2.01±0.03 | 0.01±0 | 1.08±0 | 0.64±0.01 | 3.97±0.12 | 25.17±1.13 |
| HJ19A | 25.23±0.12 | 6.96±0.01 | 8.53±0.02 | 245±6.48 | 180.67±2.05 | 10.72±0.2 | 1.96±0.02 | 0.021±0.001 | 2.11±0.02 | 0.02±0 | 0.32±0.01 | 0.85±0.01 | 4.53±0.05 | 28.78±0.09 |
| TZ19A | 25.67±0.09 | 9.03±0.01 | 8.22±0.04 | 243±3.27 | 204.33±1.7 | 10.47±0.02 | 3.46±0.02 | 0.011±0.001 | 1.91±0.01 | 0.02±0 | 0.56±0.01 | 6.45±0.06 | 3.13±0.09 | 47.15±1.28 |
| BS19A | 24.67±0.09 | 8.14±0.01 | 5.97±0.05 | 237.67±2.49 | 196±5.72 | 10.5±0.03 | 3.55±0.01 | 0.007±0.002 | 1.9±0.02 | 0.03±0 | 0.47±0.01 | 0.53±0.04 | 3.87±0.09 | 23.35±0.7 |
| LH19A | 24.87±0.05 | 8.19±0 | 6.67±0.04 | 140±2.94 | 203.33±1.25 | 7.56±0.03 | 3.05±0.01 | 0.022±0.001 | 2.83±0.04 | 0.01±0 | 1.38±0.01 | 0.54±0.03 | 5.63±0.12 | 24.08±0.66 |
| BX19A | 20.37±0.05 | 8.29±0.01 | 6.21±0.44 | 235.33±2.05 | 200.67±2.05 | 9.55±0.01 | 3.46±0.02 | 0.013±0.001 | 1.72±0 | 0.02±0 | 0.99±0.02 | 0.34±0.02 | 2.63±0.12 | 19.34±0.42 |

Table S2. Significance tests of the differences of the bacterioplankton communities.

| Compared groups | Adonis | | Anosim | |
| --- | --- | --- | --- | --- |
|  | *R^2^* | *P value* | *R* | *P value* |
| Global test | 0.4805 | **0.001** | 0.7397 | **0.001** |
| 2018S vs 2018A | 0.339 | **0.001** | 0.6737 | **0.001** |
| 2018S vs 2019S | 0.2036 | **0.021** | 0.3417 | **0.001** |
| 2018S vs 2019A | 0.4653 | **0.001** | 0.766 | **0.001** |
| 2018A vs 2019A | 0.469 | **0.001** | 0.726 | **0.001** |
| 2018A vs 2019S | 0.132 | **0.005** | 0.4193 | **0.001** |
| 2019S vs 2019A | 0.5276 | **0.001** | 0.8161 | **0.001** |

Table S3. Classification and relative abundance of module hub and connector nodes in the bacterioplankton networks.

| Samples | OTU | *Zi* | *Pi* | Generalists | Relative abundance (%) | Phylum | Class | Order | Family | Genus |
| --- | --- | --- | --- | --- | --- | --- | --- | --- | --- | --- |
| 2018S | OTU2563 | -0.1 | 0.653 | Connectors | 0.67±0.735 | Actinobacteria | Actinobacteria | Microtrichales | Ilumatobacteraceae | CL500-29_marine_group |
|  | OTU3223 | 1.75 | 0.653 | Connectors | 3.324±2.44 | Actinobacteria | Actinobacteria | Microtrichales | Ilumatobacteraceae | CL500-29_marine_group |
|  | OTU1049 | -0.793 | 0.666 | Connectors | 0.003±0.01 | Bacteroidetes | Bacteroidia | Cytophagales | Spirosomaceae | Pseudarcicella |
|  | OTU3534 | -0.75 | 0.666 | Connectors | 0.645±1.918 | Bacteroidetes | Bacteroidia | Flavobacteriales | Flavobacteriaceae | Flavobacterium |
|  | OTU3798 | 0.079 | 0.642 | Connectors | 0.187±0.288 | Bacteroidetes | Bacteroidia | Chitinophagales | norank_o__Chitinophagales | norank_f__norank_o__Chitinophagales |
|  | OTU2938 | -0.377 | 0.625 | Connectors | 0.002±0.007 | Cyanobacteria | Oxyphotobacteria | Chloroplast | norank_o__Chloroplast | norank_f__norank_o__Chloroplast |
|  | OTU2425 | 3.016 | 0.47 | Module hubs | 0.01±0.031 | Gemmatimonadetes | Gemmatimonadetes | Gemmatimonadales | Gemmatimonadaceae | Gemmatimonas |
|  | OTU1780 | 3.363 | 0.459 | Module hubs | 0.003±0.007 | Proteobacteria | Gammaproteobacteria | Cellvibrionales | Cellvibrionaceae | Cellvibrio |
| 2018A | OTU1765 | -0.291 | 0.694 | Connectors | 0.002±0.004 | Actinobacteria | Actinobacteria | Microtrichales | Microtrichaceae | IMCC26207 |
|  | OTU89 | -0.372 | 0.632 | Connectors | 0.004±0.016 | Bacteroidetes | Bacteroidia | Chitinophagales | Chitinophagaceae | Dinghuibacter |
|  | OTU3309 | 0.397 | 0.709 | Connectors | 0.008±0.02 | Cyanobacteria | Oxyphotobacteria | Chloroplast | norank_o__Chloroplast | norank_f__norank_o__Chloroplast |
|  | OTU12 | -0.423 | 0.666 | Connectors | 0.002±0.005 | Proteobacteria | Gammaproteobacteria | Oceanospirillales | Halomonadaceae | Halomonas |
|  | OTU3233 | -1.324 | 0.625 | Connectors | 0.048±0.205 | Verrucomicrobia | Verrucomicrobiae | Chthoniobacterales | Chthoniobacteraceae | LD29 |
| 2019S | OTU3748 | -0.5 | 0.625 | Connectors | 0.089±0.162 | Bacteroidetes | Bacteroidia | Flavobacteriales | Flavobacteriaceae | Flavobacterium |
|  | OTU3770 | 2.559 | 0.485 | Module hubs | 0.003±0.009 | Bacteroidetes | Bacteroidia | Bacteroidales | Prevotellaceae | Prevotella_9 |
|  | OTU4243 | 3.908 | 0.447 | Module hubs | 0.005±0.019 | Bacteroidetes | Bacteroidia | Flavobacteriales | Flavobacteriaceae | Flavobacterium |
|  | OTU1931 | 3.33 | 0.301 | Module hubs | 0.007±0.019 | Proteobacteria | Alphaproteobacteria | Rhizobiales | Rhizobiaceae | Shinella |
|  | OTU2817 | -1.432 | 0.64 | Connectors | 0.029±0.031 | Proteobacteria | Gammaproteobacteria | Betaproteobacteriales | TRA3-20 | norank_f__TRA3-20 |
|  | OTU3044 | 3.137 | 0.381 | Module hubs | 0.01±0.024 | Proteobacteria | Gammaproteobacteria | Betaproteobacteriales | Rhodocyclaceae | Dechloromonas |
| 2019A | OTU2082 | -0.929 | 0.72 | Connectors | 0.006±0.009 | Actinobacteria | Actinobacteria | Solirubrobacterales | Solirubrobacteraceae | Conexibacter |
|  | OTU3717 | -0.547 | 0.687 | Connectors | 0.017±0.051 | Actinobacteria | Actinobacteria | Microtrichales | Ilumatobacteraceae | CL500-29_marine_group |
|  | OTU2641 | -0.912 | 0.74 | Connectors | 0.002±0.007 | Firmicutes | Bacilli | Bacillales | Family_XII_o__Bacillales | Exiguobacterium |
|  | OTU1976 | 2.122 | 0.719 | Connectors | 0.023±0.061 | Patescibacteria | Saccharimonadia | Saccharimonadales | norank_o__Saccharimonadales | norank_f__norank_o__Saccharimonadales |
|  | OTU2034 | 0.697 | 0.625 | Connectors | 0.008±0.021 | Proteobacteria | Gammaproteobacteria | Betaproteobacteriales | TRA3-20 | norank_f__TRA3-20 |
|  | OTU960 | 3.02 | 0.095 | Module hubs | 0.561±0.78 | Proteobacteria | Gammaproteobacteria | Betaproteobacteriales | Methylophilaceae | Candidatus_Methylopumilus |


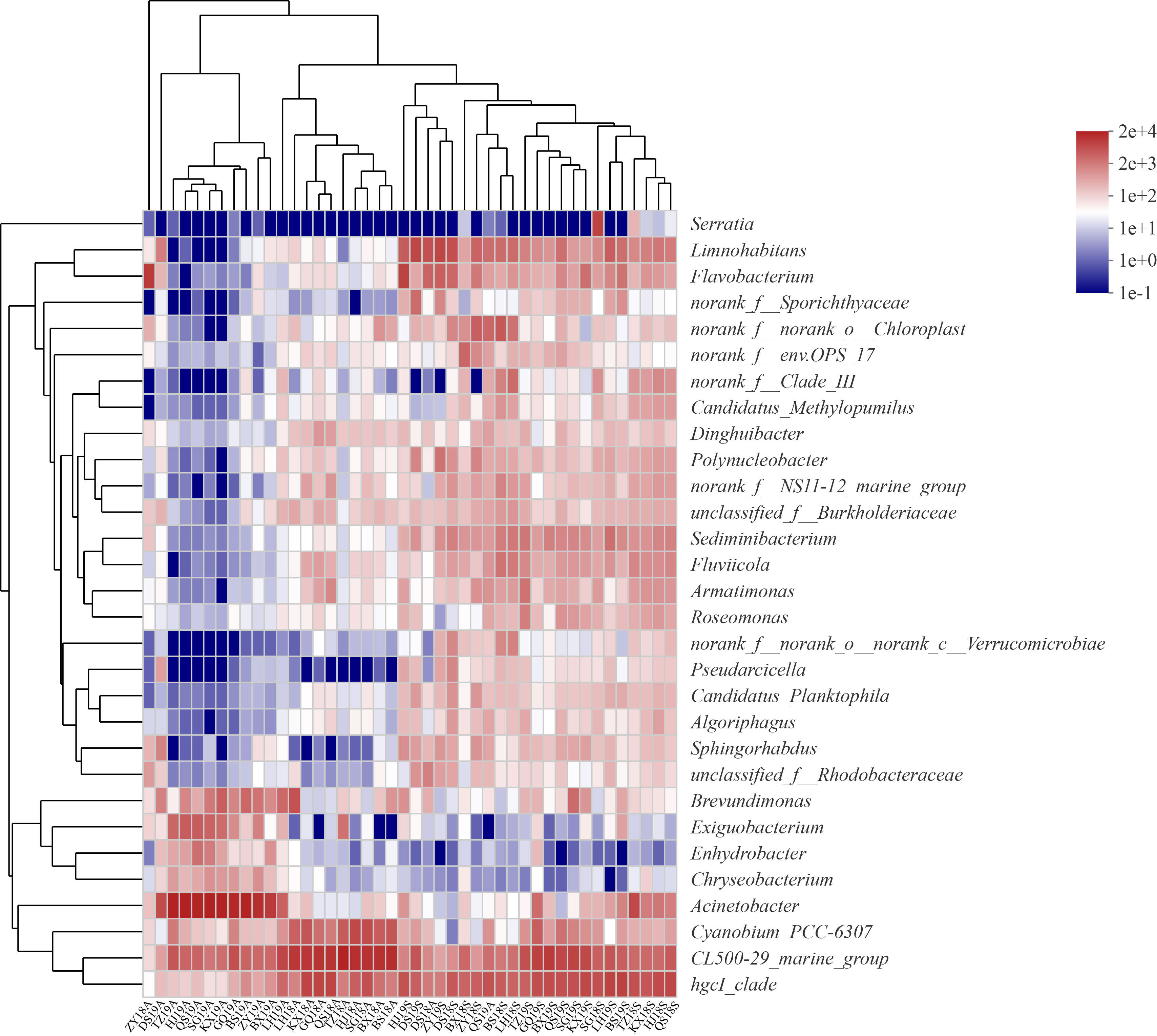
Figure S1. Heat map of the top 30 genera between bacterioplankton communities.
